# Conserved mode of nuclear lamina distortion by primate cytomegaloviruses: importance of the pSer22 motif, viral kinase and cis/trans isomerase Pin1 activity

**DOI:** 10.1099/jgv.0.002160

**Published:** 2025-10-31

**Authors:** Kishore Dhotre, Martin Schütz, Sofia von Essen, Lucio Fortelny, Christina Wangen, Friedrich Hahn, Heinrich Sticht, Manfred Marschall

**Affiliations:** 1 Harald zur Hausen Institute of Virology, Friedrich-Alexander University of Erlangen-Nürnberg (FAU), Erlangen, Germany1; 2 Division of Bioinformatics, Institute of Biochemistry, Friedrich-Alexander University of Erlangen-Nürnberg (FAU), Erlangen, Germany

**Keywords:** cytomegalovirus replication, nuclear lamina distortion, peptidyl-prolyl *cis*/*trans* isomerase Pin1, regulatory viral kinases, site-specific lamin phosphorylation, viral nuclear egress

## Abstract

Human cytomegalovirus (HCMV) is a ubiquitous human pathogen of high clinical relevance. In terms of pathogenic determination, the regulatory factors of HCMV–host interaction play a crucial role, and recently we reported on virus-supportive functions of the cellular peptidyl-prolyl *cis*/*trans* isomerase Pin1. Notably, Pin1 is able to recognize phosphorylated serine/threonine-proline motifs and regulate the structural conformation, stability and function of its substrate proteins. During HCMV replication, Pin1 facilitates viral nuclear egress by inducing site-specific rearrangements of the nuclear lamina through the *cis*/*trans* conversion of lamin type A/C. To this end, we developed readout systems to decipher details of HCMV–Pin1 regulatory interaction. Notably, together with primary human foreskin fibroblasts (HFFs) and recombinant lamin-modified cell populations, the molecular mechanisms of Pin1 interaction with both the nuclear lamina and viral proteins were illustrated. Our new results demonstrate the following: (i) currently available Pin1-inhibitory small molecules, similar to the antiviral drug maribavir (MBV), exert an antiviral activity against human and non-human primate cytomegaloviruses (CMVs); (ii) site-specific phosphorylation at serine 22, a Pin1 recognition motif within lamin A/C, is consistently mediated by the pUL97 kinase homologs of these viruses; (iii) the phosphorylation of serine 22 is sensitive to the virus-specific kinase inhibitor MBV; (iv) a doxycycline-inducible expression of autofluorescent lamin A/C-red fluorescent protein (RFP) fusion constructs in HFFs supports the productive HCMV replication; (v) these lamin A/C-RFP reporter cells indicated a virus-induced formation of lamina-depleted areas (LDAs), dependent on serine 22 but independent of the infecting CMV species; and (vi) treatment of CMV-infected cells with kinase or Pin1 inhibitors exerted distinct effects on the magnitude of LDA formation. Combined, the study is consistent with our concept that the mode of nuclear egress shows parallels between human and non-human primate CMVs. Thus, the role of Pin1 may play an important regulatory role in determining virus infection and replication efficiency.

**Impact Statement**

Mechanistic aspects of the multifaceted process of viral nuclear egress have been investigated for HCMV in the past and still have to be addressed for their conservation or variability among other cytomegaloviruses. Comparative analyses have been limited to date, so this study aimed to provide insights into this mode of virus–host interaction, as examined for three different cytomegalovirus species (HCMV, ChCMV, and RhCMV), especially including the depiction of nuclear lamina distortion and the involvement of the cellular peptidyl-prolyl cis/trans isomerase Pin1. Significant results were achieved for questions of virus-induced upregulation of Pin1, the Pin1-modulated changes of the nuclear lamina in response to CMV infections, a quantitative comparative determination of the analyzed CMVs, and the impact of inhibitory small molecules on these events. In essence, we state that the mode of nuclear egress is conserved among human and non-human primate CMVs involving viral kinase activity and the host factor Pin1 as an important regulatory determinant.

## Data Summary

**Fig. 1.**
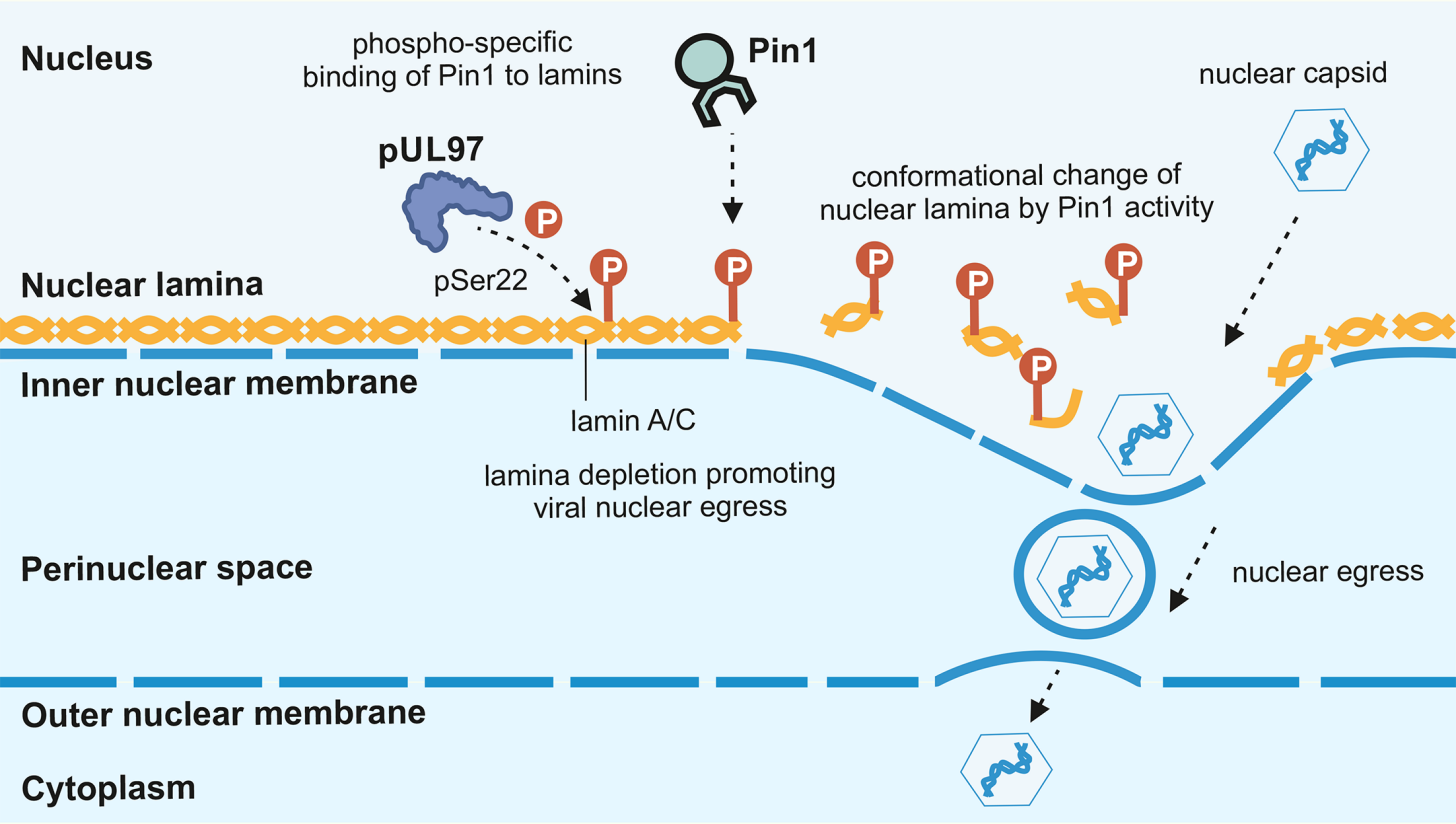
Schematic representation of the regulatory function of cellular *cis*/*trans* isomerase Pin1 during HCMV infection. During the course of HCMV infection, Pin1 may regulate several steps of virus–host interaction. Notably, Pin1 interacts with nuclear lamin A/C phosphorylated at pSer22, which significantly contributes to nuclear lamina distortion (i.e. induction of LDAs), and therefore is considered to facilitate viral nuclear egress. Thus, the process of nucleocytoplasmic transport of the viral capsids is assisted by Pin1 through LDA formation, which provides the local disassembly of the nuclear lamina required for capsid transition.

**Fig. 2.**
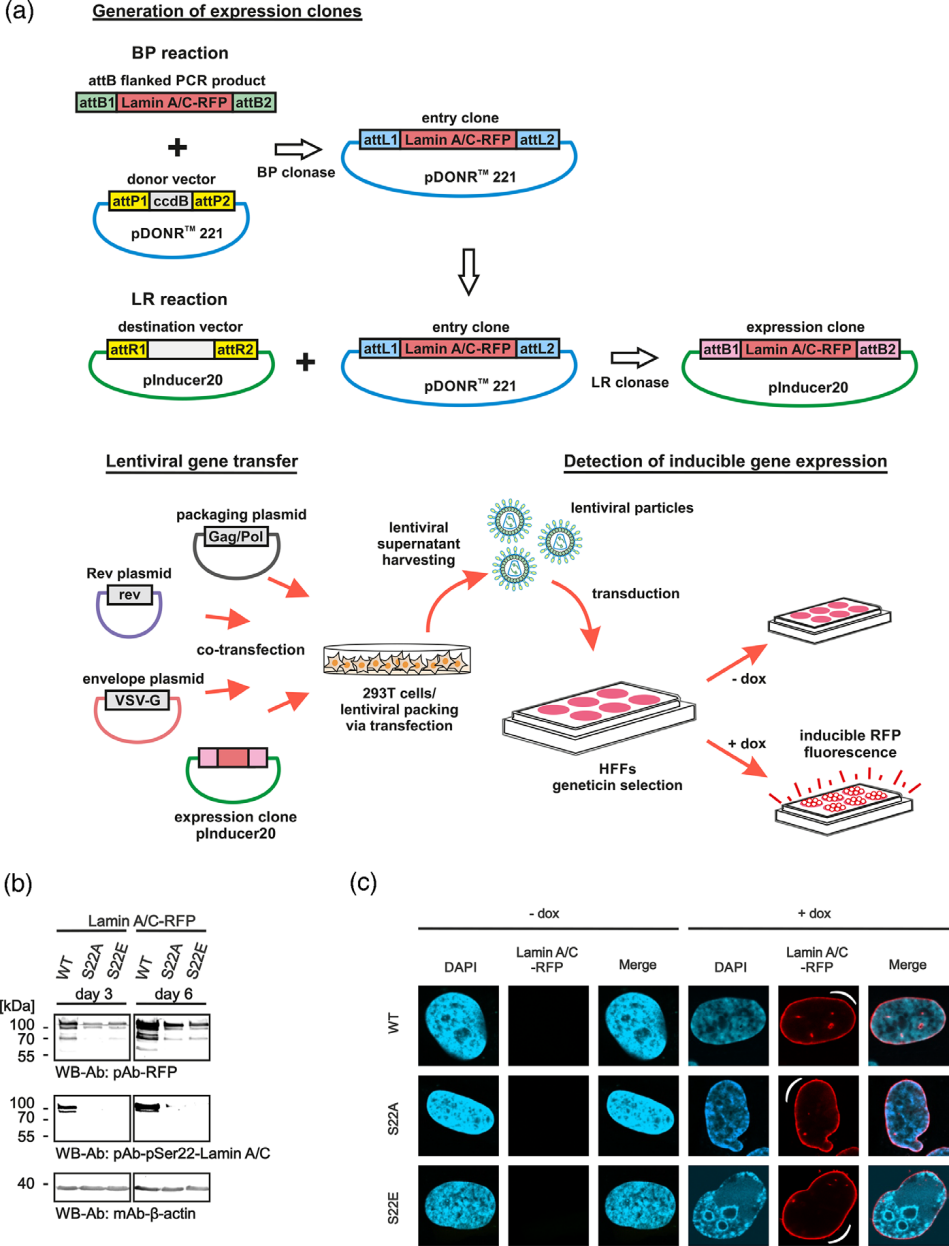
Generation of dox-inducible recombinant HFFs expressing lamin A/C-RFP by lentiviral gene transfer. (**a**) A schematic representation of steps involved in the production of recombinant HFFs expressing lamin A/C-RFP WT, phosphoserine-deficient (S22A) and phospho-mimetic (S22E) mutants. The expression cassette of lamin A/C-RFP [23] was cloned into the vector pDONR^™^ 221, based on a BP reaction using BP clonase, and then transferred into pInducer20, using LR clonase as described elsewhere (Gateway^®^ technology, Invitrogen). Lentiviral gene transfer was performed on the basis of cotransfection of 293 T cells with the packaging, rev and envelope plasmids, together with the expression clone. From these cells, lentiviral particles were harvested with media supernatant samples and utilized to transduce HFFs for subsequent geneticin selection. (**b**) Induction with dox (+dox, 500 ng ml^−1^) was performed in lamin A/C-RFP WT, S22A and S22E HFF populations, in which expression was confirmed by producing total cell lysates at 7 days post-induction, subsequently analyzed by SDS-PAGE/Wb. (**c**) For confocal imaging, the lamin A/C-RFP cell populations WT, S22A and S22E were uninduced (−dox, left panel) or induced with dox (+dox, right panel), resulting in autofluorescence based on the expressed RFP fusion construct (see white semicircular marks). The cells were fixed at 6 days post-induction and analyzed under the microscope using counterstaining of cell nuclei with DAPI. Upon +dox induction, lamin A/C-RFP expression was detected in a range of 20–60% of positive cells, dependent on the selected subpopulation.

**Fig. 3.**
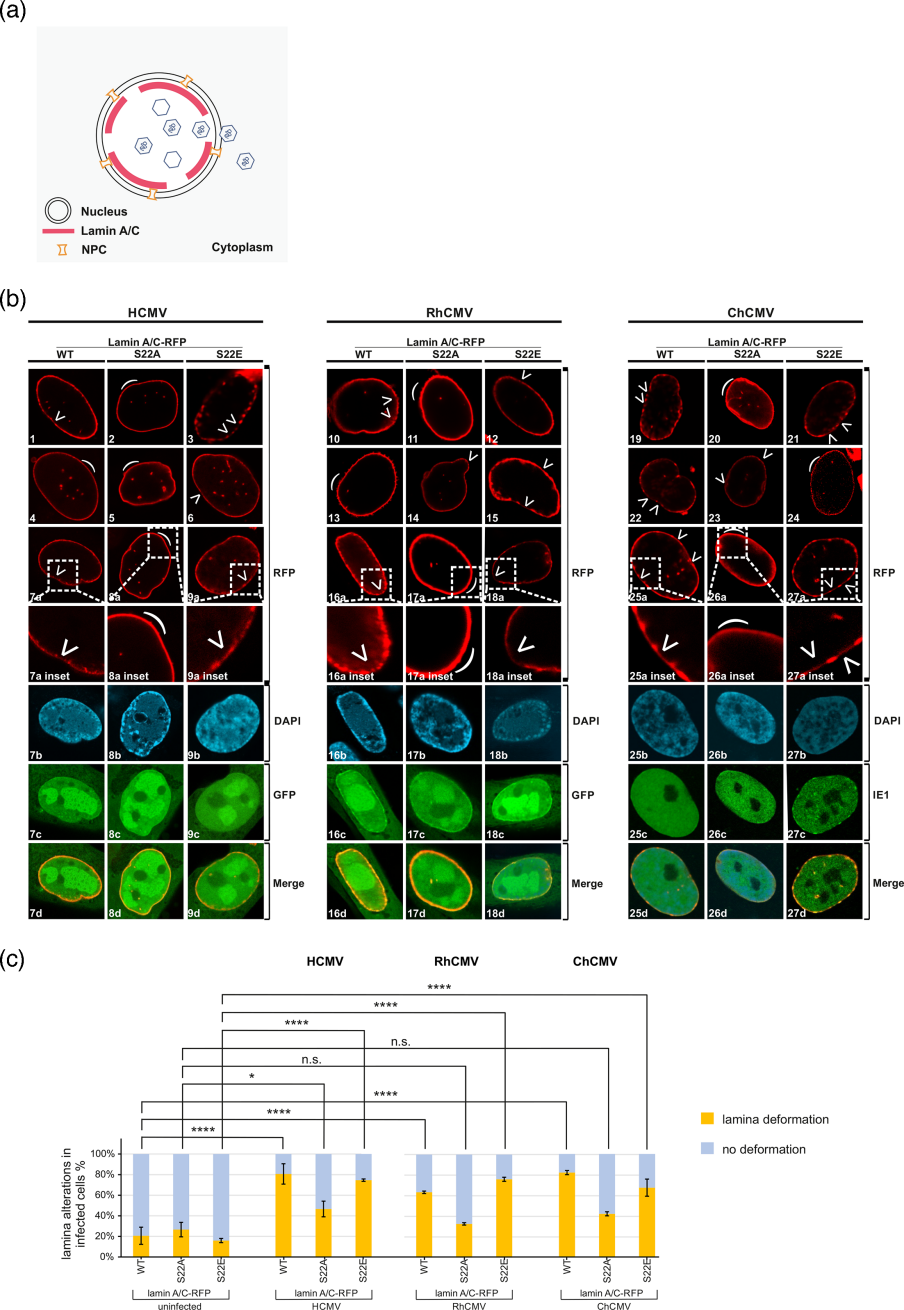
Induction of nuclear LDAs in HCMV/RhCMV/ChCMV-infected dox-induced recombinant lamina A/C-RFP fibroblasts. (**a**) Visual representation of virus-induced nuclear lamina depletion marked by LDAs to assist viral nuclear egress. (**b**) A lentiviral gene transfer procedure was applied to generate HFF populations expressing lamin A/C-RFP WT, S22A or S22E. Upon induction with dox, lamin A/C-RFP is expressed. These recombinant HFFs were seeded on glass cover slips and infected with HCMV AD169-GFP, RhCMV-GFP and ChCMV at an m.o.i. of 1. Cells were fixed at 4 d p.i., followed by counterstaining of cell nuclei with DAPI. For HCMV and RhCMV, positive GFP signals were used to confirm infection, while for ChCMV, IE1-immunostaining was used. Samples were analyz66ed by confocal microscopy (see white semicircular marks for intact nuclear lamina). (**c**) Quantitation of virus-specific nuclear lamina deformation (see white arrowheads) was performed by counting 50 cells per (biological triplicate). Statistical significance was compared with mock and infected cells for each lamin mutant using one-way ANOVA. The number of asterisks signifies the level of significance, i.e. *, *P*≤0.05; **, *P*≤0.01; ***, *P*≤0.001; ****, *P*≤0.0001; n.s., not significant, *P*>0.05.

**Fig. 4.**
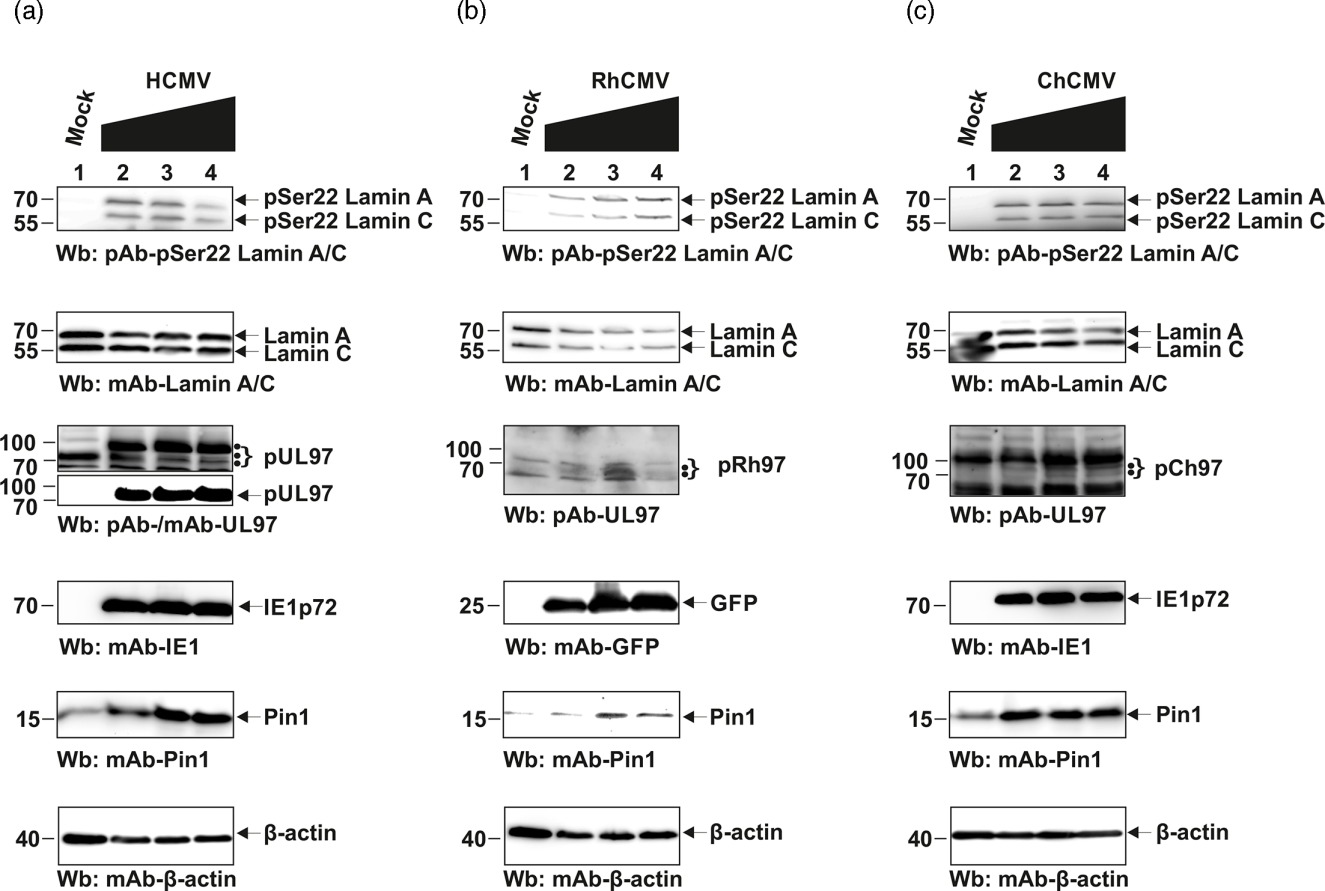
Site-specific phosphorylation of lamin A/C at Ser22 in CMV-infected primary fibroblasts analyzed by Wb detection. HFFs were infected with (a) HCMV, (b) RhCMV and (c) ChMV or remained mock-infected. Increasing m.o.i.s were applied as indicated in a range between ~0.5 and 2 for HCMV, 1 and 3 for ChCMV and 1 and 4 for RhCMV. Cells were lysed at 4 d p.i. (HCMV, RhCMV and ChCMV). Expression levels of viral marker proteins for HCMV and ChCMV IE1 (IE1p72) and RhCMV (GFP), as well as cellular lamin A/C (total or pSer22-specific) and Pin1, were analyzed by Wb using the following antibodies: mAb-IE1p72, mAb-GFP, mAb-UL97, pAb-UL97, pAb-lamin A/C pSer22, mAb-lamin A/C, mAb-Pin1 and mAb-*β*-actin. (•) represent isoforms of pUL97 and their homologs.

**Fig. 5.**
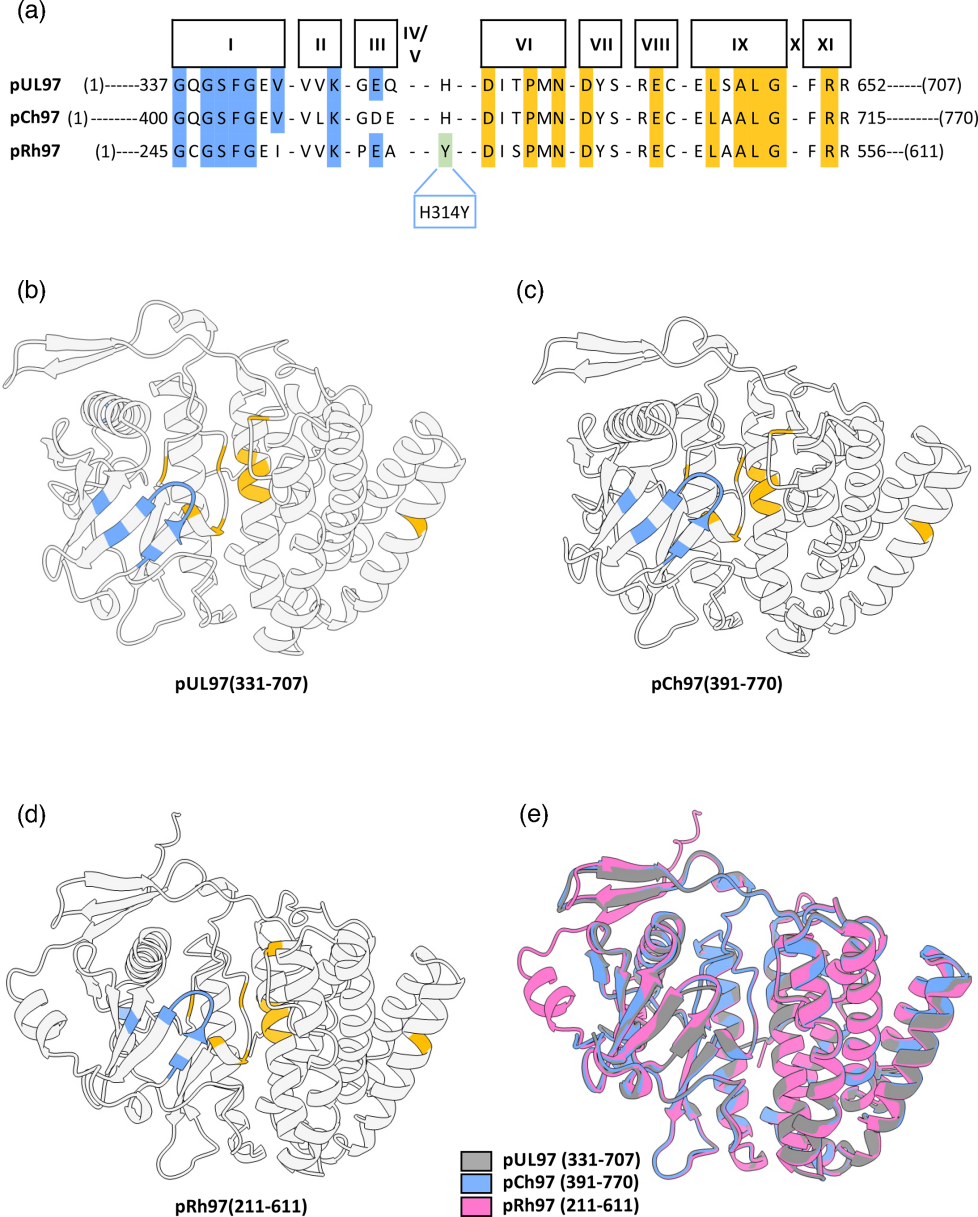
Protein sequence and structural analysis of HCMV pUL97, ChCMV pCh97 and RhCMV pRh97 globular kinase domains. (**a**) Multiple sequence alignment of pUL97, pCh97 and pRh97 illustrating the conservation of the protein kinase SDs I to XI. The SDs from I to III are represented in light blue shade and SDs from VI to XI in gold shade. The multiple sequence analysis also reveals an H314Y substitution within the kinase domain of pRh97, which corresponds to Y411 in pUL97. (**b–d**) Schematic representation of modelled structure of pUL97(331-707), pCh97(391-770) and pRh97(211-611). The residues shown in light blue and gold illustrate the conserved SDs I–III and VI–XI, respectively. (**e**) Schematic representation of superimposed pUL97(331-707) (grey), pCh97(391-770) (light blue) and pRh97(211-611) (pink).

**Fig. 6.**
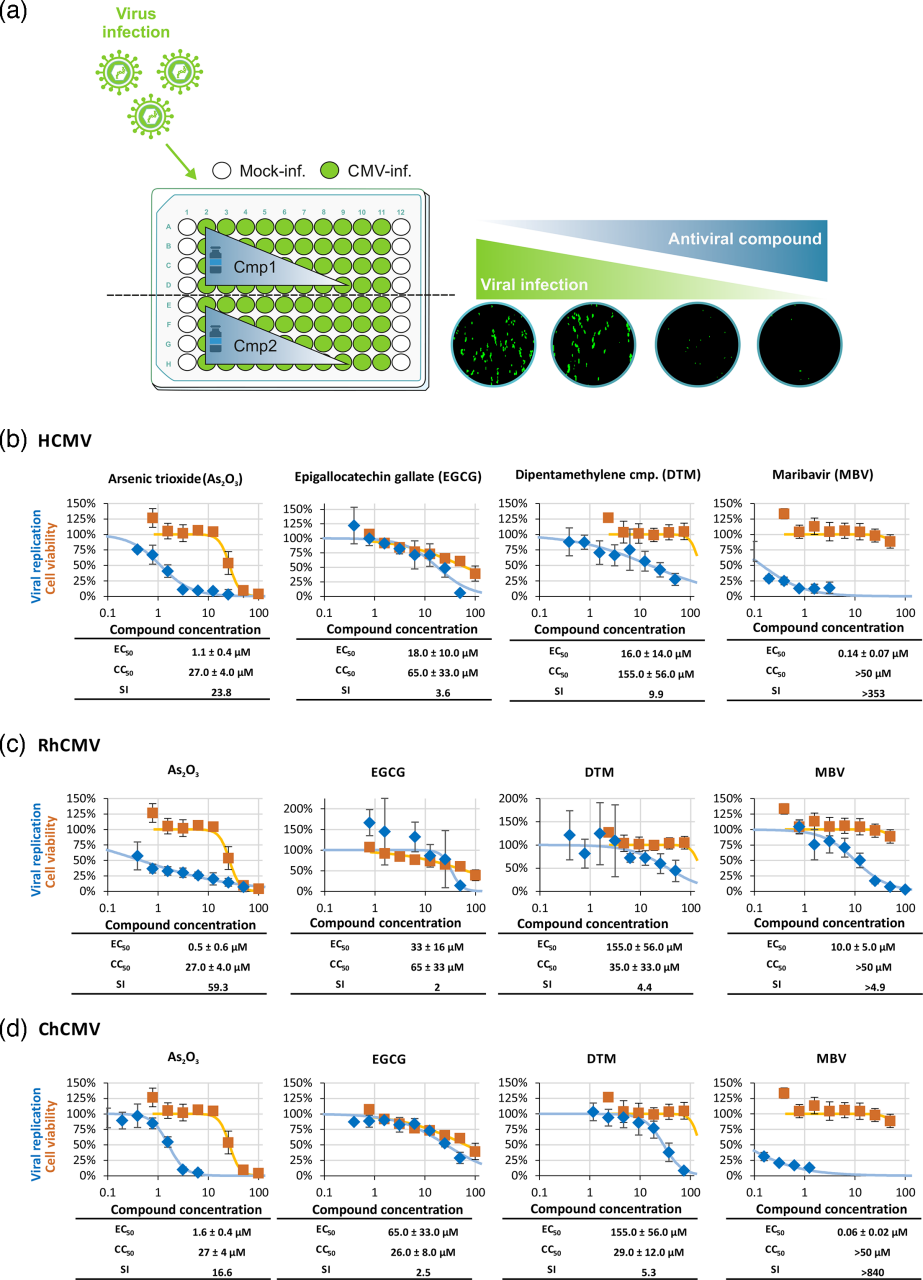
Antiviral activity of Pin1 and viral kinase inhibitors against human, rhesus monkey and chimpanzee CMVs. Primary HFFs were cultivated in a 96-well plate, and along with compound treatment, the cells were infected with HCMV AD169-GFP at m.o.i. of 0.25, ChCMV at m.o.i. of 0.5 or RhCMV-GFP at m.o.i. of 0.2 (i.e. resulting in ~25% of cells positive for GFP or virus-induced CPE, measured at 7 d p.i.). Signal quantitation was performed in quadruplets for (**a**) HCMV using GFP-based replication assay, (**b**) ChCMV using fluorescence-based IE1-immunostaining and (**c**) RhCMV using GFP-based replication assay.

**Fig. 7.**
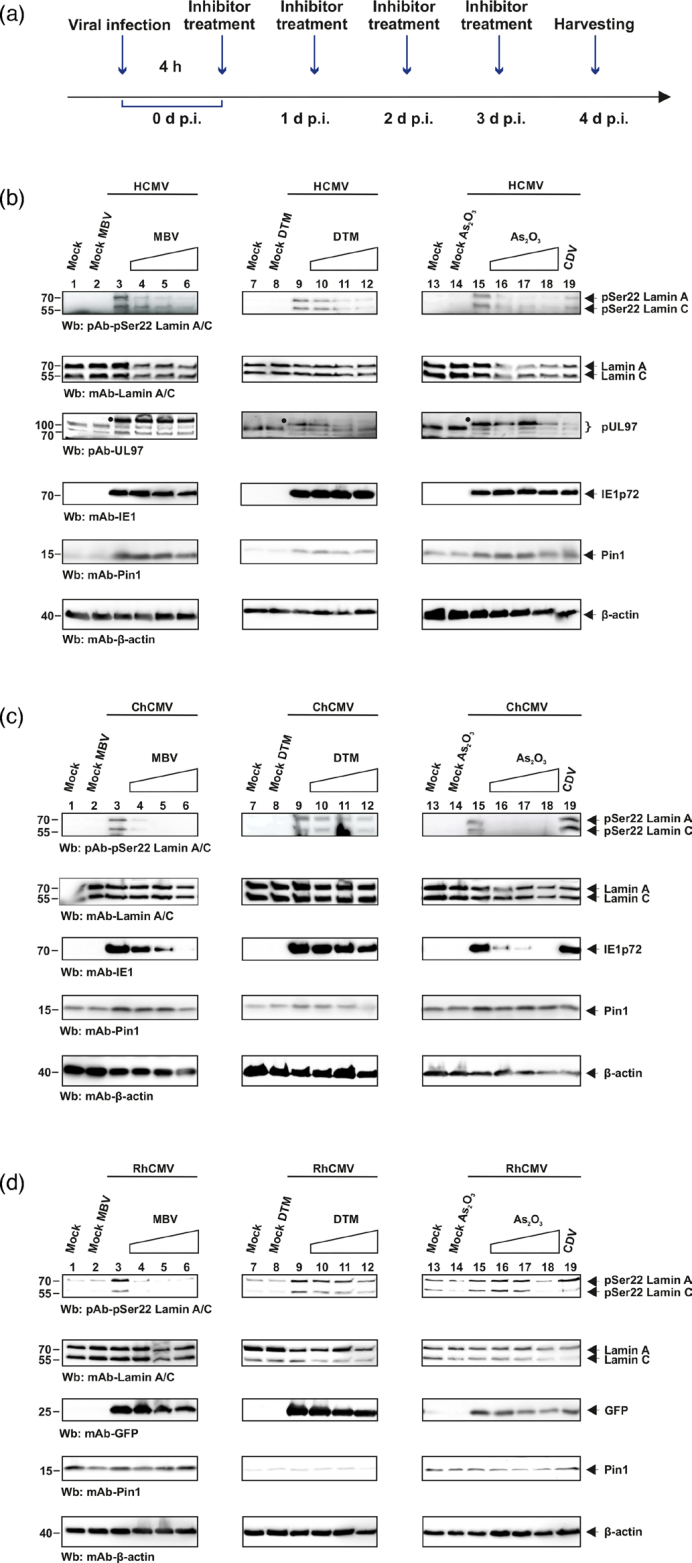
Effect of inhibitors on the site-specific phosphorylation of lamin A/C at Ser22 in herpesvirus-infected primary fibroblasts analyzed by Wb detection. (**a**) Time course of inhibitor treatment during the experiment before harvesting. Primary HFFs were infected with (**b**) HCMV strain AD169, (**c**) RhCMV-GFP and (**d**) ChCMV at m.o.i. of 1 or remained mock-infected, and at 4 h p.i., cells were treated with arsenic trioxide (As_2_O_3_; 1.5/3/6 µM), DTM (5/10/20 µM), MBV (0.7/0.14/0.28 µM for HCMV and ChCMV compared to 5/10/20 µM for RhCMV) and CDV (0.8 µM) every subsequent day. Cells were lysed at 4 d p.i. (HCMV, RhCMV and ChCMV). Expression levels of viral marker proteins for HCMV and ChCMV IE1 (IE1p72) and RhCMV (GFP), as well as cellular lamin A/C (total or pSer22-specific) and Pin1, were analyzed by Wb using the following antibodies: mAb-IE1p72, mAb-GFP, pAb-pSer22, mAb-lamin A/C, mAb-Pin1 and mAb-*β*-actin.

**Fig. 8.**
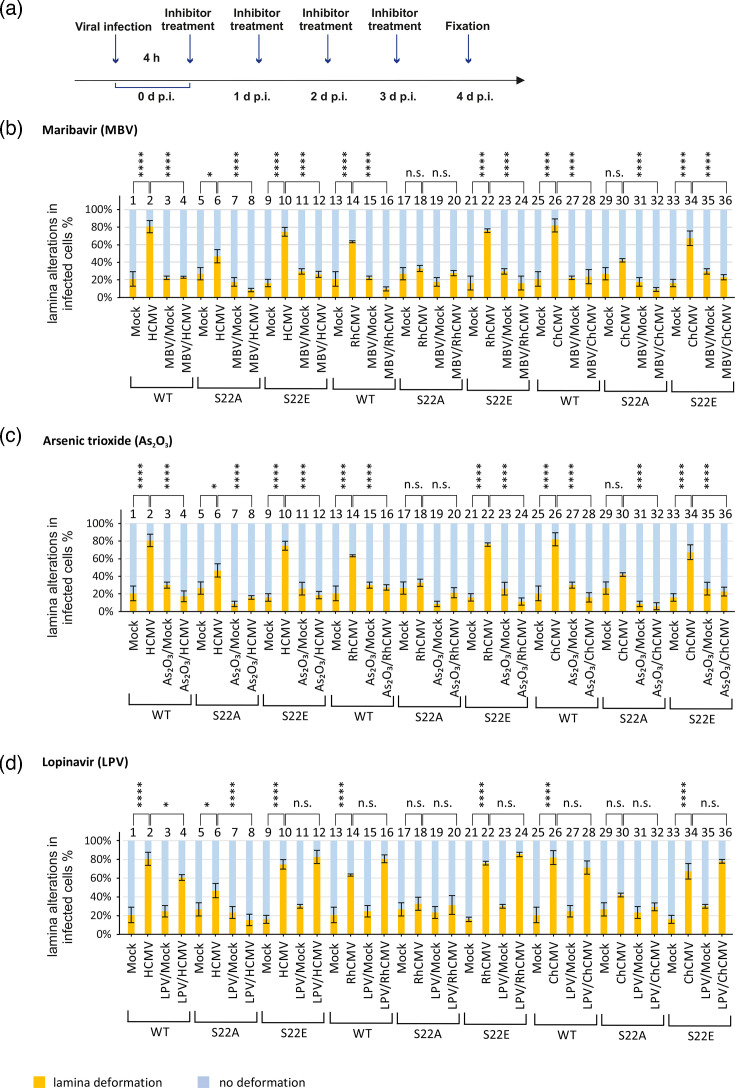
Quantitative determination of inhibitor effects on nuclear lamina deformation. (**a**) Time course of inhibitor treatment during the experiment before fixation. HFF populations expressing lamin A/C WT, S22A or S22E mutants were infected with HCMV AD169-GFP, RhCMV-GFP or ChCMV at m.o.i. of 1 or remained mock-infected. At 4 h p.i., cells were treated with MBV, As_2_O_3_ or LPV, applying daily drug refreshment: (**b**) pUL97 kinase inhibitor MBV (0.28 µM for HCMV and ChCMV; 20 µM for RhCMV); (**c**) Pin1 inhibitor As_2_O_3_ (6 µM); (**d**) negative control inhibitor LPV (7.6 nM). Quantitation of virus-specific nuclear lamina deformation was performed by counting 50 cells per biological triplicate. Statistical significance was compared with mock and infected cells for each lamin mutant using one-way ANOVA. The number of asterisks signifies the level of significance, i.e. *, *P*≤0.05; **, *P*≤0.01; ***, *P*≤0.001; ****, *P*≤0.0001; n.s., not significant, *P*>0.05.

The authors, K.D., M.S., S.v.E., L.F., C.W., F.H., H.S. and M.M., confirm that all supporting data, code and protocols have been provided within the article or through supplementary data files. Data shown by Figs 1–8 of this report illustrate the experimental findings obtained for a comparison between the modes of nuclear egress studied for the viruses HCMV, ChCMV and RhCMV, including molecular details of nuclear lamina distortion and effects referring to lamina *cis*/*trans* isomerization mediated by Pin1. The supporting external data shown by Figs S1–S6 provide accessory information for isoforms of viral protein kinases pUL97, pRh97 and pCh97.

## Introduction

Infections with the human cytomegalovirus (HCMV) are of immense clinical relevance due to the fact that HCMV is a ubiquitous human pathogen associated with various symptoms. The seropositivity in the human population ranges between 40 and 95%, depending on socioeconomic parameters. HCMV infection of immunocompetent hosts is normally asymptomatic, while infections of immunonaive or immunocompromised individuals can lead to severe symptomatic courses and even life-threatening outcomes. In particular, the acute primary HCMV infection during pregnancy represents the most critical virus-based risk to the unborn and newborn in developed countries. Viral pathogenesis is closely linked to a complex network of host interactions [1], and HCMV-directed preventive measures are still under development [2]. The HCMV permissiveness of infected tissues and the pathogenic course of productive HCMV replication are determined by a variety of virus-supportive regulatory host cell processes [3,6]. As a hallmark of HCMV–host interaction, a number of multiregulatory, mixed protein complexes are formed, composed of viral and cellular constituents. Such multiprotein complexes have been described for several well-characterized stages of HCMV replication, such as virion entry, nucleocytoplasmic capsid trafficking, nuclear egress complex, cytoplasmic assembly complex and others [7,11].

The related *β*-herpesviruses, i.e. cytomegaloviruses of rhesus macaque, chimpanzee and human (RhCMV, ChCMV and HCMV), share double-stranded, large DNA genomes, with sizes of more than 200 kbp. The viral core genes are highly conserved in sequences among cytomegaloviruses (CMVs). For example, HCMV sequence identity ranges from 60 to 90% with ChCMV and from 50 to 82% with RhCMV [12,14]. Apart from coding sequences, CMVs share numerous similarities in biological properties. As a common feature, CMVs use similar mechanisms to modulate the host regulatory and metabolic machinery for driving the efficiency of viral replication. Capsid assembly and genomic encapsidation take place in the cell nucleus, and further transport into the cytoplasm facilitates advanced steps of virion maturation. Due to the limitation that the exclusion size of the nuclear core complex is significantly smaller than nuclear capsids, the viral nuclear egress and maturation require specific mechanisms that are fulfilled by the viral nuclear egress complex [11,15,17]. For NEC-mediated regulation, it is pertinent to consider the egress-limiting role of the cellular nuclear lamina, which is an intermediate type of the cytoskeleton [11,18]. In particular, nuclear lamin A/C possesses important functions in the maintenance of the nucleus framework, restricted exchange of macromolecules, transcription regulation and in the course of mitosis [19]. During mitosis, lamin A/C undergoes area-specific rearrangement and disassembly in a way similar to herpesviral infection [20,21]. Hereby, site-specific phosphorylation of nuclear lamins is a hallmark of structural integrity and dynamics [22,23]. In response to lamin A/C phosphorylation, especially at the motif position of serine 22 (pSer22), the peptidyl-prolyl *cis*/*trans* isomerase Pin1 has been identified as one of the potential main players of nuclear lamina structural modulation (Fig. 1) [21,23,25]. According to the current state of investigations, the functional mechanism of herpesviral nuclear egress may be conserved. Concerning primate CMVs, it appears noteworthy that one crucial key of the nuclear egress effector mechanism, namely the site-specific interaction motif for viral kinase pUL97/homologs and host Pin1 in the lamin A/C coding sequence, has been consistently found. The genetic alignments of lamin A gene (LMNA) sequences in humans, rhesus macaques and chimpanzees show conservation at the pSer22 residue and the respective Pro-X-pSer-Pro Pin1-binding motif, so that Pin1 may play a similar functional role to assist viral nuclear egress (Fig. S1, available in the online Supplementary Material). In addition to nuclear lamina-specific regulation in herpesvirus-infected cells, Pin1 recognizes viral proteins and may regulate their conformation and functionality. During HCMV replication, Pin1 exerts effects on various stages, including the direct interaction with three early regulatory proteins [26]. So far, it has remained an unanswered question whether related herpesviruses may recruit Pin1 in a similar way to exploit its protein isomerase activity for virus-specific regulation.

In this study, we compare events of nuclear egress, protein–protein interaction, lamina distortion and egress-relevant antiviral drug sensitivity among HCMV, ChCMV and RhCMV. The findings indicate that the phenomenon of lamina-depleted areas (LDAs) is conserved during the nuclear egress of primate CMVs, and Pin1 facilitates the nuclear egress by interacting with the serine 22 Pin1 motif of lamin A/C. Furthermore, their relevance for biological consequences in the similarities and differences of replicative features between these CMVs is discussed.

## Methods

### Antibodies

The following primary antibodies were used in this study: human lamin A/C, rabbit mAb-Lamin A (EPR4100; Abcam plc), Ser22-phosphorylated lamin A/C, rabbit polyclonal antibody pAb-Lamin A/C Ser22 (Cell Signaling Technology), human Pin1, mouse mAb-Pin1 (SC46660; Santa Cruz Biotechnology), human Pin1, rabbit pAb-Pin1 (10495-1-AP; Proteintech), *β*-actin, mouse mAb-*β*-actin AC-15 (Sigma-Aldrich, Steinheim, Germany), viral immediate early IE1p72, mouse mAb-IE1 (kindly provided by William Britt, Birmingham, AL, USA), GFP; mouse mAb-GFP (7.1 and 13.1; Roche Life Science), mAb-UL97.01 (kindly provided by T. Lenac and S. Jonick; Department of Histology and Embryology, University of Rijeka, Croatia, used for Wb analysis), pAb-UL97 (kindly provided by D.M. Coen, Harvard Medical School, Boston, used for Wb analysis). For Western blot (Wb) analyses, secondary HRP-conjugated anti-mouse/-rabbit secondary antibodies (Jackson ImmunoResearch) and, for indirect immunofluorescence (IF) tests, Alexa Fluor^™^ 488 (A-11008, Thermo Fisher Scientific) conjugated secondary antibodies were used.

### Cell culture and viruses

Primary human foreskin fibroblasts (HFFs) were cultivated in minimal essential medium (MEM, 21090022; Thermo Fisher Scientific, Waltham, MA, USA) and supplemented with 10% FBS (F7524; Sigma Aldrich, Steinheim, Germany), 1× GlutaMAX^™^ (35050038; Thermo Fisher Scientific, Waltham, MA, USA) and 10 µg ml^−1^ gentamicin and were maintained at 37 °C, 5% CO_2_ and 80% humidity. The selected HFF cell populations expressing red fluorescent protein (RFP) tagged lamin A/C WT, S22A and S22E were cultivated in tetracycline-negative FBS (FBS-TET-12A; Capricorn Scientific, Ebsdorfergrund, Germany) supplemented with 500 µg ml^−1^ geneticin (G418, 10131035; Thermo Fisher Scientific, Waltham, MA, USA) and, for the induction of recombinant gene expression, with 500 ng ml^−1^ doxycycline (dox), refreshed every third day. HFFs or recombinant HFFs expressing RFP-tagged lamin A/C WT, S22A and S22E mutants were used for infection with virus stocks of HCMV AD169, AD169-GFP, ChCMV strain Heberling and RhCMV-GFP strain 68-1.

### Generation of recombinant HFFs, expressing lamin A/C-RFP, by lentiviral gene transfer

The cDNA fragments of lamin A/C-RFP WT, S22A and S22E were amplified by PCR as described elsewhere [23]. All constructs were generated with attB1 and attB2 ends, according to the protocol of the manufacturer (Gateway^®^ technology, Invitrogen). For gateway entry, these PCR fragments were used for BP recombination to be introduced into the vector pDONR^™^221 (Thermo Fisher Scientific, Waltham, MA, USA) and subsequently into the tetracycline/dox-inducible lentiviral vector pInducer20 (Addgene, Watertown, MA, USA). For the production of lentiviral particles, 293 T cells were transfected with a combination of lentiviral packaging (Gag/Pol and Rev) and viral envelope (VSV-G) expression plasmids, together with pInducer20-based transfer constructs of lamin A/C-RFP WT, S22A or S22E. The culture supernatants were collected, filtered through a 0.2 µm nitrocellulose membrane and stored at −80 °C. The collected lentiviral supernatants were then used for transduction in low-passage primary HFFs and supplemented with 5 mg ml^−1^ polybrene for enhancing the efficiency of lentiviral transduction. Positively transduced cells were selected with 500 µg ml^−1^ geneticin with MEM, 10% tetracycline-negative FBS, 1× GlutaMAX^™^ and 10 µg ml^−1^ gentamicin.

### Wb analysis

Wb analysis was performed according to standard protocol described before [27,28].

### Indirect IF analysis and quantitation of virus-specific nuclear lamina deformation by confocal imaging

For IF analysis, the recombinant HFFs, expressing lamin A/C-RFP WT or mutants S22A and S22E, were seeded in six-well plates at a density of 1.8×10^5^ per well on a cover slip, then induced with dox (500 ng ml^−1^), and infected with HCMV AD169-GFP, RhCMV-GFP and ChCMV at an m.o.i. of 1 or remained uninfected. At 4 days post-infection (d p.i.), cells were fixed using 10% formalin and permeabilized before indirect IF staining as described before [29,32]. The confocal laser-scanning microscopy analysis was performed on the TCS SP5 microscope with the HCX PL APO lambda blue 63 x/NA 1.4 OIL objective. Particularly for this study, high-intensity monochromatic light sources inclusive of a 405 UV laser diode, an argon laser and a 543 HeNe laser with individual lasers at spectral ranges of 415–477, 496–540 and 553–618 were used. The signal was detected using a Leica photomultiplier tube and a highly sensitive hybrid detector, and perceived signals were visualized using Leica LAS AF software (Leica Microsystems, Wetzlar, Germany). The signals were detected to obtain images and were further processed using Photoshop CS5 (Adobe Inc., San José, CA, USA). Microscopic counting of virus-specific nuclear lamina deformation was performed (counting 50 cells in triplicate for each sample).

### Pin1 inhibitors, pUL97 kinase inhibitors and antiviral reference drugs

For the analysis of antiviral activity of various compounds, Pin1 inhibitors arsenic trioxide (As_2_O_3_; Sigma-Aldrich, Steinheim, Germany) and dipentamethylene thiuram monosulphide (DTM; Sigma-Aldrich, Steinheim, Germany), epigallocatechin gallate (EGCG) (Sigma-Aldrich, Steinheim, Germany), pUL97 kinase inhibitor maribavir (MBV; Shanghai PI Chemicals Ltd., PR China) and lopinavir (LPV; Sigma-Aldrich, Steinheim, Germany), as well as the antiviral reference drug cidofovir (CDV; Sigma-Aldrich, Steinheim, Germany), were used to prepare stock solutions in DMSO and stored at −20 °C before application in the experiments at the concentrations indicated.

### Analyses of antiviral compounds in virus replication assays and cytotoxicity standard tests

To determine the antiviral activity of Pin1 inhibitors along with pUL97 kinase inhibitors and antiviral reference drugs, HFFs were cultivated in 96-well plates (1.35×10^4^ cells per well) and infected with HCMV AD169-GFP at an m.o.i. of 0.25, ChCMV at an m.o.i. of 0.5 or RhCMV-GFP at an m.o.i. of 0.2, i.e. resulting in ~25% of cells positive for GFP or virus-induced cytopathic effect (CPE) at 7 d p.i. Antiviral compounds or DMSO, as the solvent control, were applied immediately after virus adsorption, with the addition of fresh culture media. At 7 d p.i., cells were fixed using 10% formalin and incubated for 10 min at room temperature. For the quantitation of recombinant GFP-expressing HCMV and RhCMV, an automated fluorometric determination (Victor 1420 Multilabel Reader; PerkinElmer, Springfield, IL, USA) was performed in quadruplicate, using fixed cells as described before [29,30]. For the quantitation of ChCMV, viral IE1-specific IF staining was performed in 96-well plates using the ImageXpress^®^ Pico device (Molecular Devices LLC, San Jose, CA, USA [30]). The levels of compound cytotoxicity were quantitated in uninfected HFFs by using the standard Neutral Red assay (NRA). In brief, cells were seeded in 96-well plates, incubated with compounds (using staurosporine/STP at 10 µM as a cytotoxic positive control), and a series of twofold dilutions were made to be used for NRA determination after 7 days [31].

### Structural prediction of viral protein kinases pUL97, pCh97 and pRh97

The structural prediction of pUL97(331-707) (UniProtKB accession number: P16788.1), pCh97(391-770) (GenBank accession number: AAM00735.1) and pRh97(211-611) (GenBank accession number: AUI40100.1) was performed using the AlphaFold3 server [32]. The structural analysis and visualization were carried out using UCSF ChimeraX 1.9 [33].

## Results and discussion

### Current findings adding to the conceptual picture of CMV nuclear egress

The nuclear egress of CMVs, and other herpesviruses, is a finely regulated process resulting from the concerted action of several controlling determinants. It is generally accepted in the field that the viral core NEC, which is structurally and functionally conserved, plays a crucial role in this regulation, but that additionally the associated viral and cellular proteins within the multicomponent NEC, such as kinases, isomerases, bridging factors and others [11], are additionally important. Recently, the composition of *α*-, *β*- and *γ*-herpesviral multicomponent NECs has been identified by proteomic-based analyses, thus showing both identical cross-viral features and virus-specific differences [8,34,39]. Specifically, for the *β*-herpesviruses HCMV and the murine CMV (MCMV), some identical components have been found in the form of the two core NEC proteins, the virus-encoded protein kinase (pUL97, pM97), and the host bridging factor p32/gC1qR [35,36], but also differences became obvious (e.g. endophilin A2 as a specific component of the MCMV complex). Therefore, the question remained open as to what extent the NEC formation and the regulation of nuclear egress are identical or very similar between species of CMVs. As our initial results of this study, we observed very common properties in viral replication among HCMV, ChCMV and RhCMV, which pointed to similarities in their regulation of nuclear egress. As described by the present report, these common properties refer to the following findings: (i) the microscopically visible CPEs in the host cell nuclei resulting from infection with either of the three CMVs; (ii) the expression of nuclear egress-regulating viral kinase homologs, i.e. pUL97, pCh97 and pRh97, all represented in distinguishable isoforms (in the case of HCMV pUL97, referring to M1, M74 and M157 [40,41]); (iii) the site-specific phosphorylation of nuclear lamin A/C; and (iv) CMV-induced nuclear lamina reorganization that is dependent on both the phospho-specific motif mediating binding of *cis*/*trans* isomerase Pin1 to lamin A/C and recruitment activity of the viral core NEC [23,24, 42] (Fig. 1). Details of these results are shown in the sections below (see Figs1^8^), so that, combined, a common concept about the CMV-specific nuclear egress is postulated: current information points to the overall importance of viral kinase activity, nuclear viral protein–host interaction and the events leading to localized depletion of the nuclear lamina, including Pin1-mediated *cis*/*trans* isomerization.

### Generation and characterization of a recombinant cellular model system, based on primary HFFs, to study comparative aspects of the cytomegalovirus nuclear egress

The rationale behind the generation of dox-inducible recombinant HFFs expressing RFP-tagged lamin A/C in its WT versions, compared to the S22A and S22E mutants, was based on previous findings. Data provided evidence that the phosphorylation of lamin A/C at serine 22 (i.e. motif Pro-X-pSer-Pro) is recognized by Pin1, resulting in a conformational change of the lamin A/C protein, which promotes lamina disassembly during HCMV infection, similar to related events in mitosis [31,43,45]. These constructs were expected to express RFP-tagged lamin A/C, specifically following the induction with dox, to allow for an inspection of virus-induced formation of LDAs visualized using confocal imaging. To this end, recombinant primary HFFs were generated, using the lentiviral gene transfer methodology, to generate HFF subpopulations expressing the lamin A/C-RFP construct under dox induction (Fig. 2b). Note the phospho-specific detection (pSer22) of lamin A/C-RFP WT, but not mutants S22A and S22E (central panels). The correct intracellular localization of all three versions of lamin A/C-RFP, as presented by a pronounced nuclear rim staining resulting from the insertion of recombinant protein into the endogenous nuclear lamina structure, was proven by dox-inducible RFP fluorescence (Fig. 2c, see marking by white symbols as bent curves). Notably, in the absence of dox induction, no background expression of RFP signal was observed (Fig. 2c, left part, −dox).

### Quantitative evaluation of CMV-specific nuclear lamina distortion using the recombinant lamina-reporter system

The herpesviral process of nuclear egress is based on the nuclear assembly of capsids and their encapsidation of the viral DNA genome, followed by the NEC-mediated activities of the formation of LDAs and nucleocytoplasmic capsid transition (Fig. 3a). To address whether in our recombinant system, the CMV-infected cells exhibit measurable lamina distortion during nuclear egress, a confocal imaging analysis was carried out (Fig. 3b). Recombinant HFFs were infected with HCMV AD169-GFP (left), RhCMV-GFP (middle) and ChCMV (right) at an m.o.i. of 1 for 4 days. In the case of ChCMV, viral IE1 protein was immunostained as a lytic viral marker (using HCMV IE1 antibody), instead of GFP used for HCMV AD169-GFP and RhCMV-GFP, to achieve virus-specific detection (Fig. 3b). Confocal analysis of uninfected cells revealed a predominant number of cells showing a normal phenotype of nuclear rim-shaped lamina (see bent curve symbols; compare Fig. 2c). The infection with CMVs (HCMV, RhCMV and ChCMV in similar patterns) produced a significant increase in the number of WT cells with visual LDAs and/or a thinning of the lamin A/C-RFP signal (Fig. 3b, images 1, 7/10, 6/19, 22, 25, respectively). Importantly, in cells expressing lamin mutants S22A or S22E, the comparison of infections with either of the three CMVs showed distinct staining patterns that were, in part, different to WT. Hereby, S22A remained mostly devoid of LDAs under these CMV infections (consistent with our earlier findings specific for HCMV [31], pointing to the importance of pSer22 site-specific phosphorylation (Fig. 3b, images 2, 5, 8/11, 17/20, 26). The phospho-mimetic mutant S22E, however, showed in most parts a WT-like pattern of infected-cell lamina distortion (Fig. 3b, images 3, 6, 9/12, 15, 18/21, 27). Consequently, due to the fact that in some areas of analyzed cells the lamina distortion and LDA formation were not easily detectable or appeared in variable shapes (Fig. 3b, images 4/13/24), a fine quantitation was performed by microscopic counting (Fig. 3c). Here, compared to uninfected cells (Fig. 3c, left to each CMV panel), which remained at levels below 26.7% of visible effects, the infection with either HCMV, RhCMV or ChCMV in WT cells showed a marked degree of the fraction of cells which display lamina alterations between 63.3 and 82.7%. The S22A cells, however, indicated a substantially lower nuclear lamina alteration for all three CMV infections (32.7–46.7%). Notably, the cells expressing the phospho-mimetic S22E mutant showed only marginal differences from the effects seen in WT cells, thus appearing statistically homogeneous (i.e. differences insignificant, with alterations between 67.3 and 76.0%). These findings support our notion that pSer22, as the key position of the Pin1-binding motif in lamin A/C, plays a crucial role in virus-induced lamina distortion and that in primate CMVs this regulatory aspect is conserved.

### Patterns of viral and cellular protein production, including lamin A/C pSer22 phosphorylation, in HCMV-, ChCMV- and RhCMV-infected HFFs

The state of lamin A/C phosphorylation is considered a hallmark of herpesviral nuclear egress regulation. In particular, the site-specific modification in nuclear lamin A/C serine 22 (pSer22), induced by the HCMV-encoded protein kinase pUL97, represents a rate-limiting step for nuclear lamina rearrangement and the production of LDAs. In order to investigate how CMV infections influence cellular lamin A/C, including their pSer22 intensities, on the basis of viral protein expression patterns, we performed comparative Wb analyses (Fig. 4). HFFs were infected with HCMV, RhCMV, or ChCMV at increasing m.o.i.s (ranging between m.o.i. 0.5 and 4 as indicated; Fig. 4). Infected cells were harvested at 4 d p.i., and total lysates were subjected to the Wb analysis. Total levels of lamin A/C (pAb-lamin A/C) were compared to the phosphorylated varieties by using a phospho-specific antibody (pAb-pSer22 lamin A/C; Fig. 4a–c, upper two panels). Of note, the site-specific lamin phosphorylation, i.e. pSer22 lamin A/C, was substantially upregulated through either of the three CMV infections (Fig. 4, upper panels; compare to mock-infected control samples in lane 1). The overall protein levels of lamin A/C showed either no response or only a marginal upregulation (as principally expected on the basis of our earlier data [23]). Notably, Pin1 expression increased in response to CMV infections (Fig. 4a–c, compare panels Pin1 with *β*-actin; viral proteins were stained as markers of infection using antibodies against IE1 for HCMV and ChCMV, as well as GFP for RhCMV-GFP). Thus, in the present experiments, the three analyzed CMVs showed substantial similarities in the property of lamin A/C phosphorylation.

Furthermore, we addressed the question of whether the detection of direct interaction between Pin1 and lamin A/C is reserved to its WT form, whereas the mutant versions of conditionally expressed lamin A/C might lack such signals of interaction. To this end, coimmunoprecipitation (CoIP) experiments were performed (similar to our CoIP data published before [24]). These experiments confirmed a CoIP signal for lamin A/C in the immunoprecipitates obtained with anti-Pin1 antibody, using proteins from HCMV-infected HFFs. Interestingly, only some limited or no difference in CoIP signal was detectable when comparing the lamin A/C WT with mutant versions (Fig. S2). It has to be emphasized that, under these CoIP conditions, based on total cellular lysates with native proteins, a distinction between recombinant lamin A/C-RFP and endogenously expressed WT lamin A/C is generally difficult. Although the recombinant forms can be reliably detected by the use of RFP-specific antibodies, the endogenously expressed lamin A/C WT can hardly be dissected from higher-order interactive complexes with mutant forms. Due to the fact that lamin A/C-RFP becomes incorporated into the nascent polymeric assemblies of the endogenous nuclear lamina, the CoIP approach is not perfectly differentiating between WT and mutant, thereby consistently indicating Pin1 interaction. As far as related published reports are concerned, additional points of evidence have been collected, illustrating that Pin1 and/or conformational change is essential for aspects of nuclear lamina structure and function, including the role of lamin mutations [19,46, 47]. However, the question of site-specific lamin replacement mutations, in the context of Pin1 binding, has not been specifically addressed in the previous reports.

In addition to these aspects, we also analyzed the expression patterns of viral kinase pUL97 and its homologs, all detected by pUL97-specific antibodies. Interestingly, as a consistent feature of the three CMVs, distinguishable isoforms of their kinases are expressed. In the case of HCMV, these have been designated as M1, M74 and M157 (pAb-UL97 detecting the three isoforms, upper panel; mAb-UL97 mostly detecting M1, lower panel; Fig. 4a–c, lanes 2–4) [40], and likewise, isoforms were also detected for the homologous kinases of RhCMV and ChCMV, termed pRh97 and pCh97 (Figs 4a–c, lanes 2–4 and S3). Thus, our data showed for the first time that RhCMV, ChCMV and HCMV produce isoforms of the pUL97 kinase/homologs, albeit the pattern of these isoforms was not found to be identical. Interestingly, the lack of an N-terminal portion of the coding sequence in the case of RhCMV obviously leads to a lack of the large 100 kDa isoform (termed M1 in the case of HCMV pUL97). Also, M74 is missing in pCh97, and M157 is missing in pRh97, but, on the other hand, these viruses are still able to produce N-terminally differentiated isoforms. This latter point refers to our earlier finding that, in the case of HCMV, the mutational elimination of individual methionine residues did not completely block the formation of pUL97 isoforms in recombinant expression systems ([40,48]; additional unpublished data, Marschall laboratory, 2025). This situation prompted us to rationalize the kinase expression features in the context of the 3D structure [43,45,49]. Although the overall primary sequence identity among the kinase homologs pUL97, pCh97 and pRh97 is generally low (≤45.8% identity at amino acid level; Fig. S4), there is a marked conservation of kinase subdomains (SDs) I to XI (Fig. 5a). Notably, the AlphaFold models indicated a very similar fold predicted for all three homologs (Fig. 5b–d). In the highly conserved signature sequences of kinase SDs I to XI, the structural properties appear almost identical. Differences can be specifically seen in some peripheral loop regions, which have different lengths, such as the helical loop exclusively occurring in pRh97 (Fig. 5e, left side in the overlay). Combined, the modelling suggests a highly similar fold of the pUL97, pCh97 and pRh97 globular kinase domains. Based on these structural features of pUL97 and its homologs, as well as protein expression patterns and detected viral markers together with host proteins Pin1 and lamin A/C, we were interested in the antiviral potency of inhibitors of Pin1 and pUL97 in a comparative manner.

### Establishment of anti-CMV drug assessment systems, and the antiviral efficacy of Pin1-inhibitory as well as pUL97-inhibitory small molecules

In order to assess the anti-CMV efficacy of antiviral compounds in a quantitative manner, specific test systems were established in 96-well formats based on the infection of HFFs. HCMV- and RhCMV-specific systems were performed according to the automated fluorescence measurement of the reporter GFP, and a related ChCMV-specific system had been based on the IF staining of viral IE1 protein, as described before [29,30, 52, 53]. In all cases, multiple biological and technical replicates of the settings were performed, and compound concentrations were measured in quadruplicate (Fig. 6a–d). On the one hand, the inhibitory compounds served as tools for mechanistic investigations, and, on the other hand, they may foster a novel antiviral strategy, including the application of pharmacological inhibitors against viral kinases, the host isomerase Pin1 and additional targets. Three different Pin1 inhibitors (As_2_O_3_ [54]; DTM [55]; EGCG [56]) were compared to the inhibitor of the HCMV kinase pUL97 (MBV). As a particular finding, As_2_O_3_ and DTM showed antiviral activity against all three viruses and substantially decreased viral replication signals in a concentration-dependent manner, while EGCG only exerted some partial antiviral activity (possibly due to certain differences in the mode of Pin1-inhibitory efficacy of these drugs; Fig. 6b–d). In parallel, the putative anti-CMV broadness of activity of the clinically approved pUL97 inhibitor MBV was addressed. Our data showed the expected strong activity against HCMV (0.14±0.07 µM; Fig. 6b) and also high activity against ChCMV (0.06±0.02 µM; Fig. 6d); however, a largely reduced MBV sensitivity in the case of RhCMV (10.0±5.0 µM; Fig. 6c). Interestingly, an analysis of available viral sequences identified a distinct amino acid replacement in pRh97 [57,58], i.e. H411Y, which had been described as one of the experimentally validated MBV resistance-conferring mutations in HCMV pUL97 [57,58]. Since the similarity of our structural modelling approach did not suggest any global differences in protein folding, we rather tend to correlate the measured differences in MBV sensitivity between the CMVs to a variation of individual amino acids in their kinases, in particular to a naturally occurring reduced MBV sensitivity of RhCMV based on the pRh97(H314Y) replacement (Figs 5a and and S3–S5 or 4–5). Seen apart from this distinctive feature, the antiviral drug assessment indicated that both Pin1 and pUL97 inhibitors can significantly and broadly interfere with HCMV, ChCMV and, in part, RhCMV replication, all measured in HFFs.

### Determination of the effect of Pin1 and pUL97 inhibitors on lamin A/C pSer22 levels in CMV-infected cells

The effect of distinct inhibitory small molecules on pSer22 phosphorylation in CMV-infected cells was then investigated by phospho-specific Wb analysis. To this end, HFFs were infected with either of the three CMVs (at a high m.o.i. of 1), and after the 4 h phase of virus adsorption, the cells were subjected to repeated inhibitor treatment by daily replacement of culture media with drug refreshment as indicated (Fig. 7a). CDV served as a broad antiherpesviral reference drug (Fig. 7b–d, lane 19). For pUL97 inhibitor MBV treatment, as well as the two Pin1 inhibitors DTM and As_2_O_3_, the Wb staining patterns (Fig. 7b–d) indicated at least a partial reduction of pSer22 levels of lamin A/C (lanes 4–6, 10–12, 16–18, for either of the drugs), in the case of the three CMVs. Antiviral activity was detected, mostly for the highest concentration of each drug (lanes 6, 12, 18), by partly reduced levels of viral pUL97, IE1 or reporter GFP (Fig. 7b–d, panels Wb: pAb-UL97-A1, mAb-IE1, mAb-GFP). For MBV (lanes 4–6), the inhibition of pSer22 level was strong in the case of HCMV (Fig. 7b) and ChCMV at an identical MBV concentration (Fig. 7c; 0.28 µM). But for RhCMV, the similar reduction in pSer22 signal was only observed at a higher concentration of MBV (20 µM; Fig. 7d), referring to the relatively high EC_50_ value of MBV determined for RhCMV (Fig. 6c). Interestingly, the two Pin1 inhibitors, As_2_O_3_ and DTM, produced a reduction of pSer22 in the case of HCMV and ChCMV (Fig. 7b, c, lanes 10–12, 16–18) but much lower, only to a modest degree at their highest drug concentration, in the case of RhCMV (Fig. 7d, lanes 12 and 18). Thus, the Pin1 inhibitor-produced pSer22 reduction probably was mainly due to the antiviral drug activity, which led to limited expression of the viral kinase, i.e. HCMV pUL97 or ChCMV/RhCMV homologs, respectively (Fig. 7b, panels Wb: pAb-UL97-A1, lanes 10–12, 16–18). This indicated that the inhibitor activities against pUL97 and Pin1 could not be dissected, in the way of pSer22-active versus pSer22-inactive drug effects, as originally thought in these experimental settings. This finding was in part surprising but was based on the antiviral potential of all drugs, which comprised multiple inhibitory effects, thus not allowing a statement in regard to selective pSer22 inhibition. Even when the incubation of the Pin1 inhibitor As_2_O_3_ was only started 2 d p.i. (instead of immediately p.i.), there was still a marked reduction in pSer22 in the case of HCMV, albeit some lower level of reduction in the case of ChCMV (Fig. S6). Independent from these limitations in the dissection of specific contributions to lamin modification, the approach underlined the virus-induced Ser22-specific phosphorylation of lamin A/C in cells infected with either of the three CMVs and the inhibition of this phosphorylation by the activity of the analyzed drugs.

### Treatment with both Pin1 and pUL97 inhibitors reduces LDA formation and restores the normal phenotype of lamin A/C-RFP in CMV-infected cells

Finally, a confocal imaging analysis of LDA formation in CMV-infected cells was performed, also addressing the impact of these pUL97- or Pin1-inhibitory drugs. For this purpose, HFFs were infected with either of the three CMVs (at m.o.i. of 1) before cells were subjected to inhibitor treatment (as described for Fig. 8a, by daily replacement of culture media with drug refreshment). The signal quantitation of nuclear lamina morphology indicated the previously described, pronounced virus-specific lamina deformation, including LDA induction, in WT lamin A/C-RFP cells for each of the three CMVs (Fig. 8b–d; for virus-induced upregulation of LDAs, compared to each mock-infected control, see lanes 1–2 HCMV, lanes 13–14 RhCMV and lanes 25–26 ChCMV, respectively). The known pattern of lamin A/C mutant-specific modulation of signals was again found for S22A (lanes 5–6, 17–18, 29–30) and S22E (lanes 9–10, 21–22, 33–34). Under conditions of MBV treatment (Fig. 8b), the signals of lamina deformation and LDAs were reduced substantially, in some cases to basically mock-like negative levels, for HCMV (lanes 4, 8, 12, compare mock control lane 1) and ChCMV (lanes 28, 32, 36, compare mock control lane 25) when applying an identical MBV concentration (0.28 µM), while RhCMV showed a clear reduction of LDA formation at a higher drug concentration (20 µM; lanes 16, 20, 24), referring to the relatively high EC_50_ value of MBV determined for RhCMV (Fig. 6c). With our novel, quantifiable approach, even HCMV-, RhCMV- or ChCMV-infected cells expressing the mutant S22E of lamin A/C-RFP (considered as a constitutive phospho-mimicking binding site for Pin1) showed MBV sensitivity (lanes 12, 24 and 36). This effect may result from the strict limitation of virus-induced Pin1 upregulation under the antiviral conditions of MBV treatment or may even indicate a viral induction of the Pin1 enzymatic activity required for S22E recognition, which might be suppressed under MBV treatment. An alternative explanation would be given by putative serine 392 phosphorylation, as a second target site of nuclear lamina rearrangement. Our earlier investigations [23] clearly identified Ser22 as the main site of CMV-induced lamin A/C phosphorylation, while Ser392, as another mitosis-specific phosphorylation site, has minor relevance. Actually, however, we cannot rule out the possibility that, under the given conditions of infection, there may be a shift from Ser22- to Ser392-specific phosphorylation. Moreover, concerning the treatment with the Pin1 inhibitor As_2_O_3_, we found a relatively similar pattern of inhibition as for MBV. Also, with As_2_O_3_ treatment, the signals of lamina deformation and LDAs were reduced substantially (Fig. 8c, lanes 4, 8, 12 for HCMV; lanes 16, 20, 24 for RhCMV; lanes 28, 32, 36 for ChCMV). Notably, in contrast to MBV, As_2_O_3_ treatment also showed LDA-reducing efficacy in the case of RhCMV, which is consistent with the identified strong anti-RhCMV activity of As_2_O_3_ (see Fig. 6c). As a heterologous negative control, we included the antiretroviral drug LPV, which does not exert any known inhibitory activity against CMVs (Fig. 8d). Accordingly, LPV treatment did not reduce the signals of lamina deformation or LDA induction for any of the three CMVs (lanes 3, 16, 28). In particular, LPV did not reduce signals of cells expressing the mutant S22E of lamin A/C-RFP (lanes 12, 24, 36), thus strengthening the idea that Pin1 recognition of S22E responds to CMV infection and anti-CMV inhibitors. Combined, these findings are consistent with our concept of at least a partly conserved mode of nuclear lamina distortion (as supported by the Ser22 mutants, yet not decisively traceable by the inhibitors) by the analyzed CMVs. Our current scenario emphasizes the importance of the Ser22 motif, as phosphorylated by viral kinase activity, and thus a subsequent recognition by the host *cis*/*trans* isomerase Pin1 is highly suggestive.

## Conclusions

The mechanism of nuclear egress is a rate-limiting step in the CMV replication, and the process is dynamically regulated by virus–host protein interactions. In our current study, we explored the functional relevance of nuclear egress regulators in the case of human and non-human primate CMVs, particularly focusing on the impact of Pin1 in nuclear lamina distortion by HCMV, ChCMV and RhCMV. Our findings further suggest an involvement of Pin1 in the nuclear egress mechanism, although the use of inhibitors could not fully dissect the role of Pin1 from that of the viral kinase pUL97. The underlying regulatory processes appear to be at least in part conserved among CMVs. These definitely involve the CMV-encoded protein kinases and site-specific lamin A/C phosphorylation. As indicated by earlier reports, this sequence of events may then involve the protein *cis*/*trans* isomerization by the recruited cellular peptidyl-prolyl isomerase Pin1 [23,24, 26, 59]. Specifically, we generated dox-inducible, selected populations of primary HFFs that express a dox-inducible fluorescent version of lamin A/C integrated into the host cell nuclear envelope. Applying these, we were able to bring to light the partly conserved, partly differential phenotypes of primate CMVs in nuclear lamina rearrangement and the respective sensitivity to inhibitory small molecules. Main findings were provided for the importance of residue serine 22 (S22) in lamin A/C. Upon infection with primate CMVs, the formation of LDAs was significantly and consistently increased in the case of cells expressing lamin A/C-RFP WT (WT/S22) or the phospho-mimetic (S22E) mutant. In contrast, cells expressing the phosphoserine-deficient mutant (S22A) did not produce significant LDAs upon infection with any of the three CMVs. This finding of a conserved mode of nuclear lamina rearrangement substantiated our understanding of the interaction among nuclear lamin A/C, viral protein kinases and cellular Pin1. In essence, our study provides new experimental indications of a mode of nuclear egress that may in specific points be shared between primate CMVs, as based on a phosphorylation-triggered, Pin1-dependent distortion of the host nuclear lamina. This process obviously provides one of the determinants of viral lytic replication efficiency. Future studies may additionally illustrate the involvement of even more regulatory cofactors contained in viral multicomponent nuclear egress complexes and in the mechanistic fine-diversification of herpesviral egress pathways.

## Supplementary material

10.1099/jgv.0.002160 Uncited Supplementary Material 1.

## Abbreviations

CDV: cidofovir
CMVs: cytomegaloviruses
CoIP: coimmunoprecipitation
CPE: cytopathic effect
dox: doxycycline
d p.i.: days post-infection
DTM: dipentamethylene thiuram monosulphide
EGCG: epigallocatechin gallate
HCMV: human cytomegalovirus
HFFs: human foreskin fibroblasts
IF: immunofluorescence
LDAs: lamina-depleted areas
LPV: lopinavir
MBV: maribavir
MCMV: murine CMV
MEM: minimal essential medium
NRA: Neutral Red assay
RFP: red fluorescent protein
SDs: subdomains
Wb: Western blot
