# Supplementary material for: Conserved mode of nuclear lamina distortion by primate cytomegaloviruses: importance of the pSer22 motif, viral kinase and cis/trans isomerase Pin1 activity
Source: J Gen Virol. 2025 Oct 31;106(10):002160. doi: 10.1099/jgv.0.002160 (PMC12578131; doi:10.1099/jgv.0.002160)
Supplement: Uncited Supplementary Material 1. [file jgv-106-02160-s001.pdf]

Supplementary Material

Conserved mode of nuclear lamina distortion by primate cytomegaloviruses: importance of the pSer22 motif, viral kinase, and cis/trans isomerase Pin1 activity

Kishore Dhotre[a], Martin Schütz[a§], Sofia von Essen[a], Lucio Fortelny[a], Christina Wangen[a], Friedrich Hahn[a$], Heinrich Sticht[b] & Manfred Marschall[a]

[a]Harald zur Hausen Institute of Virology, Friedrich-Alexander University of Erlangen- Nürnberg (FAU), Erlangen, Germany; [b]Division of Bioinformatics, Institute of Biochemistry, FAU, Erlangen, Germany

[§]Present address: La Jolla Institute for Immunology, San Diego, CA, USA

[$]Present address: Institute of Virology, Ulm University Medical Center, Ulm, Germany

Supplementary Figures

Pin1 binding motif

Pro-X-pSer-Pro

	1	2	3	4	5	6	7	8	9	10	11	12	13	14	15	16	17	18	19	20	21	22	23	24	25	26	27	28	29	30
LMNA_HUMAN	M	E	T	P	S	Q	R	R	A	T	R	S	G	A	Q	A	S	S	T	P	L	S	P	T	R	I	T	R	L	Q
LMNA_RHESUS	M	E	T	P	S	Q	R	R	A	T	R	S	G	A	Q	A	S	S	T	P	L	S	P	T	R	I	T	R	L	Q
LMNA_CHIMPANZEE	M	E	T	P	S	Q	R	R	A	T	R	S	G	A	Q	A	S	S	T	P	L	S	P	T	R	I	T	R	L	Q

Fig. S1. The Pin1 binding motif is conserved in the lamin A/C of humans, rhesus macaques, and chimpanzees. The illustration represents the N-terminal 1-30 amino acid sequence of human, rhesus macaques, and chimpanzee pre-lamin A/C. A multiple sequence alignment (MSA) of the pre-lamin A/C amino acid sequence of human (Uniprot accession number: P02545), rhesus macaques (F7GLE9), and chimpanzee (H2Q091) was performed using MEGA X software. The MSA analysis shows that the Pin1 binding motif 20Pro-21X-22pSer-23Pro in lamin A/C is conserved among humans, rhesus macaques, and chimpanzees.

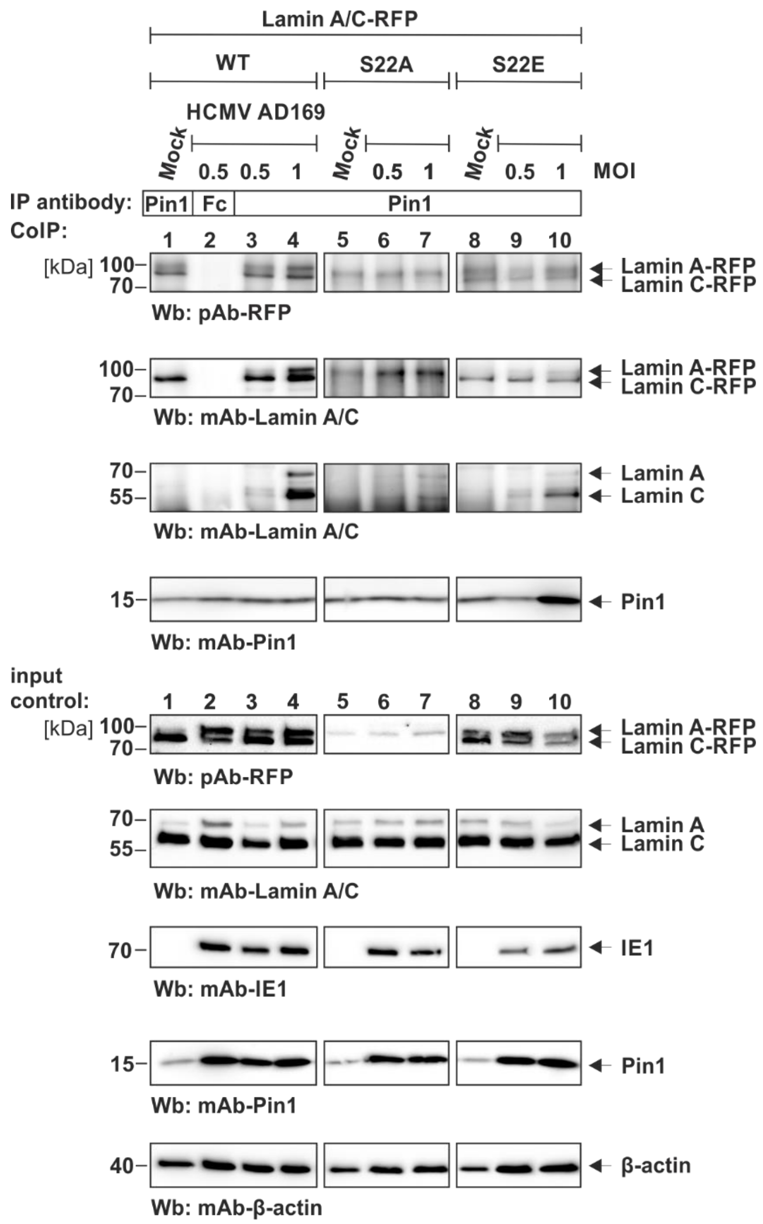

Fig. S2. Interaction analysis of Pin1 with lamin A/C, using Lamin A/C-RFP-expressing cells infected with HCMV AD169. HFF populations, expressing lamin-A/C WT, S22A, or S22E, were seeded in a T-175 cell culture flask at a seeding density of 3.6 x 10^6. On the next day, cells were infected with HCMV AD169 at MOI of 0.5 or 1, or remained uninfected (mock). At 4 d p.i., the cells were lysed, and lysates were used for coimmunoprecipitatation of lamin A/C together with the endogenous Pin1, using 3 µl of anti-human Pin1 pAb (10495-1-AP, Proteintech; lanes 1, 3-10), or the Fc fragment as a negative control (Dianova 011-000-008; lane 2). Input control samples were taken prior to CoIP, and Western blot analysis was performed with both, CoIP and input control samples, using specific antibodies as indicated (mAb-Lamin A/C for endogenous and recombinant RFP-fused versions of lamin A/C; pAb-RFP for recombinant Lamin A/C-RFP; mAb-Pin1 for endogenous Pin1; mAb-β-actin used as a loading control).

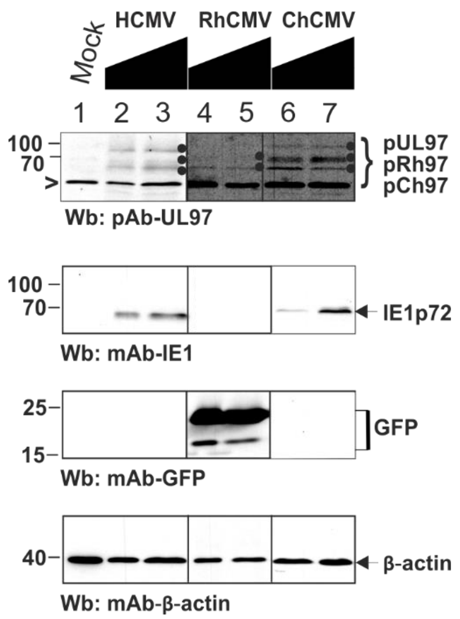

Fig. S3. Western blot detection of the viral protein kinase pUL97, and its homologs pRh97 and pCh97. HFFs were infected in a 12-well format with HCMV AD169, RhCMV-GFP, and ChCMV at an increasing MOIs, or remained uninfected. At 7 d p.i the cells were harvested and lysed for protein analysis by SDS-PAGE and Western blot using antibodies against pUL97, IE1, β-actin, and GFP. (•), represent detected isoforms of pUL97 and their homologs and (>) represents cross-reactive band.

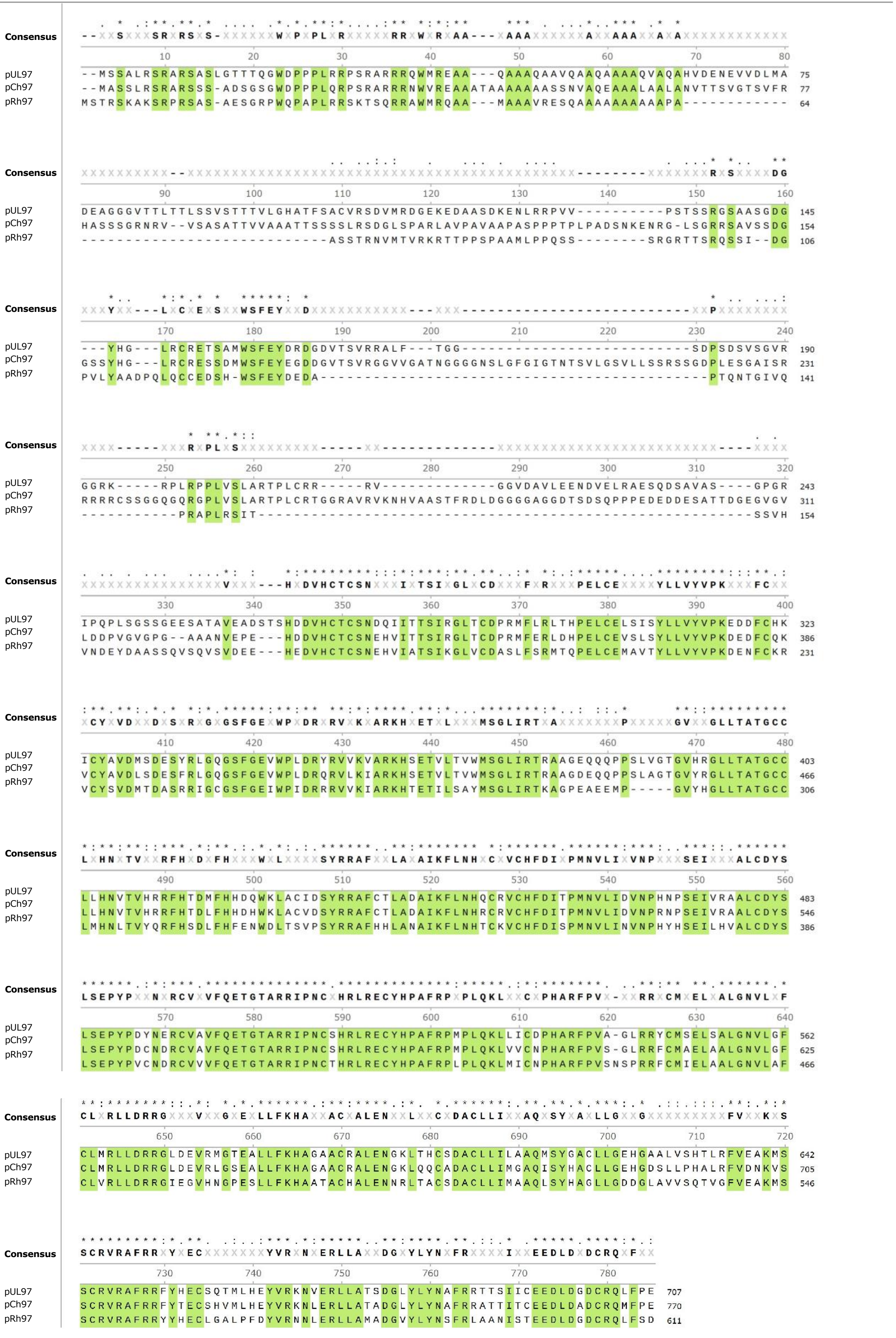

Fig. S4. Multiple sequence alignment of viral protein kinase pUL97, pCh97, and pRh97 amino acid sequences. The alignment highlights conserved amino acids in colour green; *, represents ≥ 95% amino acid sequence identity.

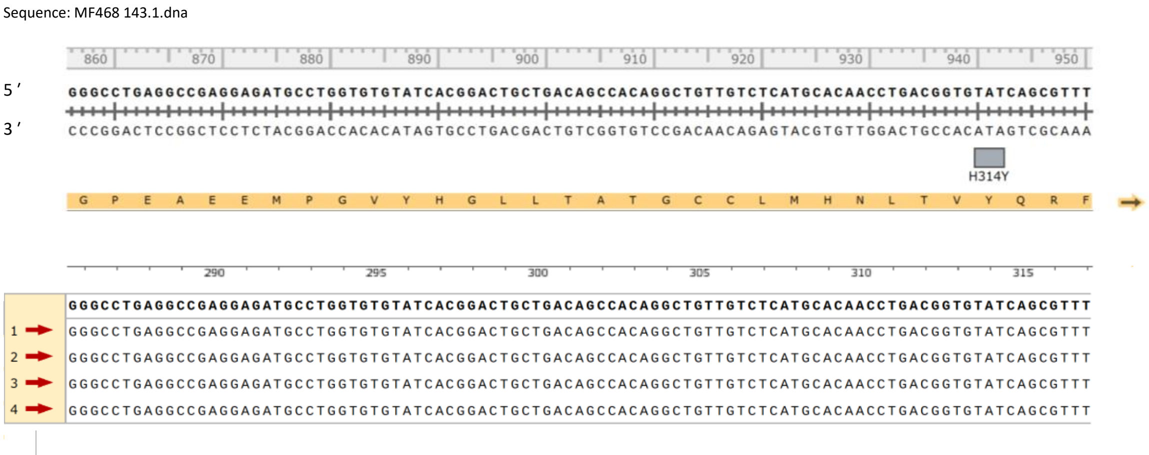

Original Sequence: MF468143.1.dna

1: f733_F_2487ZAE037_premix

2: f733_F_2487ZAE038_premix

3: f394_F_2487ZAE035_premix

4: f394_F_2487ZAE036_premix

Fig. S5. Pairwise sequence alignment. An alignment of the reference coding sequence of viral protein kinase pRh97 (GenBank accession number: MF468143.1) with PCR amplicons using sequencing primers was done to validate the existence of amino acid exchange H314Y, which correlates with the reduction in MBV sensitivity.

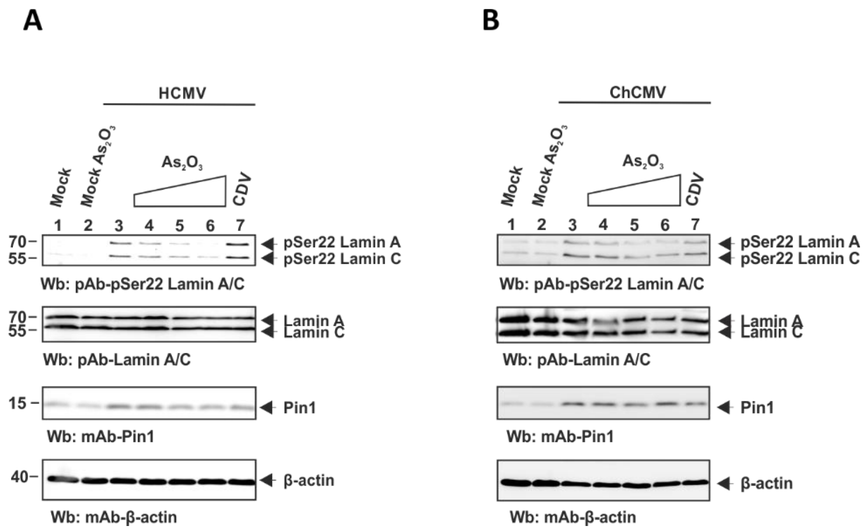

Fig. S6. Effect of inhibitors on the site-specific phosphorylation of lamin A/C at Ser22 in herpesvirus-infected primary fibroblasts analyzed by Western blot detection. Primary HFFs were infected with **(A)** HCMV strain AD169 **(B)** ChCMV at MOI of 1, or remained mock-infected. At 2 d p.i., cells were treated with arsenic trioxide (As_2O_3). Cells were lysed at 4 d p.i., and total lysates were used for the monitoring of the expression levels of cellular lamin A/C (total or pSer22-specific) and Pin1 by Western blot analysis, using the indicated antibodies (pAb-pSer22, pAb-lamin A/C, mAb-Pin1, and mAb-β-actin).
